# TAASRAD19, a high-resolution weather radar reflectivity dataset for precipitation nowcasting

**DOI:** 10.1038/s41597-020-0574-8

**Published:** 2020-07-13

**Authors:** Gabriele Franch, Valerio Maggio, Luca Coviello, Marta Pendesini, Giuseppe Jurman, Cesare Furlanello

**Affiliations:** 1 Fondazione Bruno Kessler, Trento, Italy; 2 University of Trento, Trento, Italy; 3 University of Bristol, Bristol, UK; 4 Meteotrentino, Trento, Italy; 5 HK3 Lab, Milan, Italy

**Keywords:** Computational science, Atmospheric dynamics

## Abstract

We introduce TAASRAD19, a high-resolution radar reflectivity dataset collected by the Civil Protection weather radar of the Trentino South Tyrol Region, in the Italian Alps. The dataset includes 894,916 timesteps of precipitation from more than 9 years of data, offering a novel resource to develop and benchmark analog ensemble models and machine learning solutions for precipitation nowcasting. Data are expressed as 2D images, considering the maximum reflectivity on the vertical section at 5 min sampling rate, covering an area of 240 km of diameter at 500 m horizontal resolution. The TAASRAD19 distribution also includes a curated set of 1,732 sequences, for a total of 362,233 radar images, labeled with precipitation type tags assigned by expert meteorologists. We validate TAASRAD19 as a benchmark for nowcasting methods by introducing a TrajGRU deep learning model to forecast reflectivity, and a procedure based on the UMAP dimensionality reduction algorithm for interactive exploration. Software methods for data pre-processing, model training and inference, and a pre-trained model are publicly available on GitHub (https://github.com/MPBA/TAASRAD19) for study replication and reproducibility.

## Abstract (summary)

**Table:** 

Measurement(s)	hydrological precipitation process
Technology Type(s)	computational modeling technique • digital curation
Factor Type(s)	year of data collection
Sample Characteristic - Environment	climate system
Sample Characteristic - Location	Autonomous Region of Trentino-Alto Adige/Sudtirol

Machine-accessible metadata file describing the reported data: 10.6084/m9.figshare.12529484

## Background & Summary

Effects of climate change on the increased frequency and magnitude of extreme weather events have been consistently described^1,2^, and a shift towards a more extreme precipitation climate (the so-called “tropicalisation”) has been predicted by models^3^ and observed across Europe^4,5^. In this work, we focus on precipitation *nowcasting*, i.e. forecasting within a short time interval (e.g. 0 to 6 hours), in particular for extreme and fast evolving precipitation events^6^.

State of the art solutions for precipitation nowcasting are solidly based on weather radar data^7^, due to a known direct relationship between radar reflectivity and rain rate^8^. Significant efforts have been undertaken to share open source weather radar resources, including software for analysis and visualization^9–14^ and open data repositories (see https://openradarscience.org/opendata/). Open datasets are collected and maintained by international Weather Data institutions across the US and Europe. The main publicly available products are (a) RADOLAN and RADKLIM^15^, by the German Weather Service; (b) NEXRAD Level II^16^, by the US National Oceanic and Atmospheric Service (NOAA); and (c) the dataset by the Royal Netherlands Meteorological Institute (KNMI)^17^. All these datasets provide radar reflectivity (as well as rain-gauge) data, with 1 km spatial resolution, and 5 min temporal resolution.

Here we release TAASRAD19, a dataset of weather radar maps covering an area of 240 km of diameter, collected in the Trentino South Tyrol region, in the center of the Italian Alps. TAASRAD19 features more than 9 years (7/2010–12/2019) of reflectivity product of the radar, at high spatial resolution (0.5 km) with 5 min temporal updates. TAASRAD19 is the first available resource for sub-kilometer, high-frequency, extended time-span weather data for the Italian Alps. Notably, highly variable orography and environmental complexity make precipitation forecasting exceptionally challenging in the area. The temporal coverage of the dataset (almost 900 thousand time steps over about 1,250 days with precipitations) is thus a key enabler for developing computational models for precipitation nowcasting and early detection of extreme events, in particular for implementing analog ensemble models and machine learning solutions.

The dataset has been released on Zenodo, providing the full collection of all time steps^18,19^, along with a curated selection of 1,732 sequences of precipitation (362,233 time steps)^20,21^. The sequences describe a wide range of precipitation events, including extreme rain phenomena, exceptional downpours, long intense snowfalls, and localized hailstorms. Events have been annotated by experts with precipitation classification tags extracted from daily weather summaries. As a technical validation of TAASRAD19, the annotated data are used to develop a deep learning solution for precipitation nowcasting^22^. Finally, the structure of each image data can be explored with an interactive data visualization of an Uniform Manifold Approximation and Projection (UMAP) embedding^23^. The UMAP dimensionality reduction method can be used for unsupervised machine learning analysis^24^; on TAASRAD19, it has been used in combination with a generalization of Dynamic Time Warping distance^25^ to implement fast analog ensemble search among radar sequences^26^.

## Methods

**Fig. 1:**
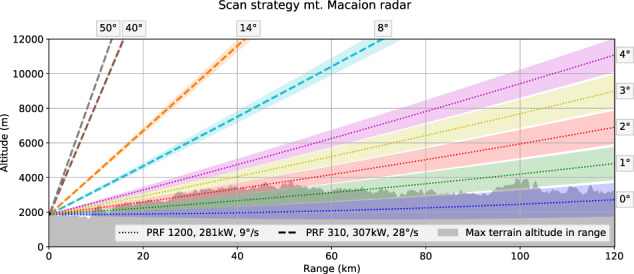
Scan strategy and signal characteristics of the mt. Macaion radar. Different Pulse Request Frequency (PRF), Power and rotation speeds are used in low and high elevation scans. Low elevation scans perform slower rotation (9°/*s*), use higher pulse frequency (1200) and lower power (281 kW), while high elevation scans perform fast rotation (28°/*s*), use low pulse frequency (310) and higher power (307 kW). The maximum terrain altitude for each range bin is reported in gray, showing that substantial beam blocking is encountered at lower elevations (0–2 degrees).

The data included in TAASRAD19 were provided by *Meteotrentino*, the official Civil Protection Weather Forecasting Agency of the Autonomous Province of Trento, Italy. The agency operates a single-polarization Doppler C-Band Radar, in collaboration with *Meteobolzano*, the Civil Protection Agency of the Autonomous Province of Bolzano. The latter is responsible for the maintenance, operation and calibration of the receiver, as well as the generation of the products, while Meteotrentino is responsible for all the downstream tasks, *i.e* quality control, rainrate conversion, forecasting and alerting. The radar is located on Mt. Macaion (1,866 m.a.s.l.), within a complex orographic environment in the center of the Italian Alps (N 46 29′18″, E 11 12′38″). The radar system is an EEC DWSR-2500C and has been in operation since 2001 at the beginning with different operating modes and scan strategies (6 to 10 minutes time-steps). Between 2009 and 2010 the radar analog receiver was upgraded with the installation of an Eldes NDRX digital receiver. The update has improved both the signal quality and the scanning frequency of the radar system. Since the upgrade completed in mid 2010, the radar has been operating with the same scan strategy at a constant time-step of 5 minutes, for a total of 288 time steps per day. Details about the operational parameters and scan strategy are reported in Table 1 and Fig. 1 respectively.

**Fig. 2:**
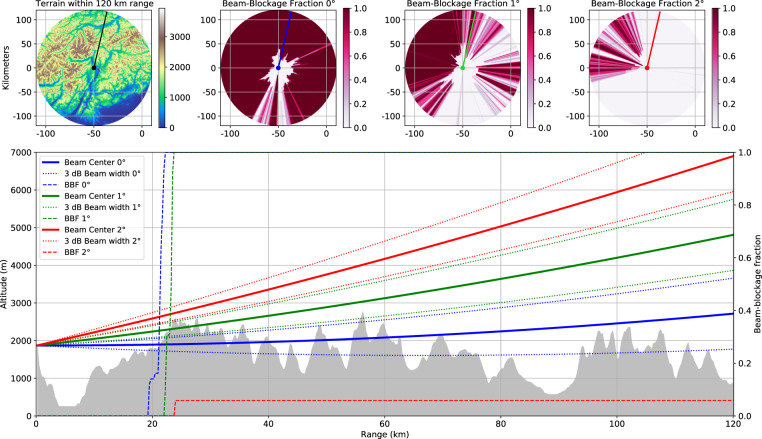
Beam Blockage Fraction (BBF) at different range and altitude. The upper row shows the digital terrain model, and the beam blockage percentage for the scans at 0, 1 and 2 degrees of elevation. The blue, green and red segments at 13 degrees azimuth on the maps (above) correspond to the cross section lines shown in the lower plot. The dashed lines represent the Beam Blockage Fraction (BBF) for each elevation at a given range.

**Fig. 3:**
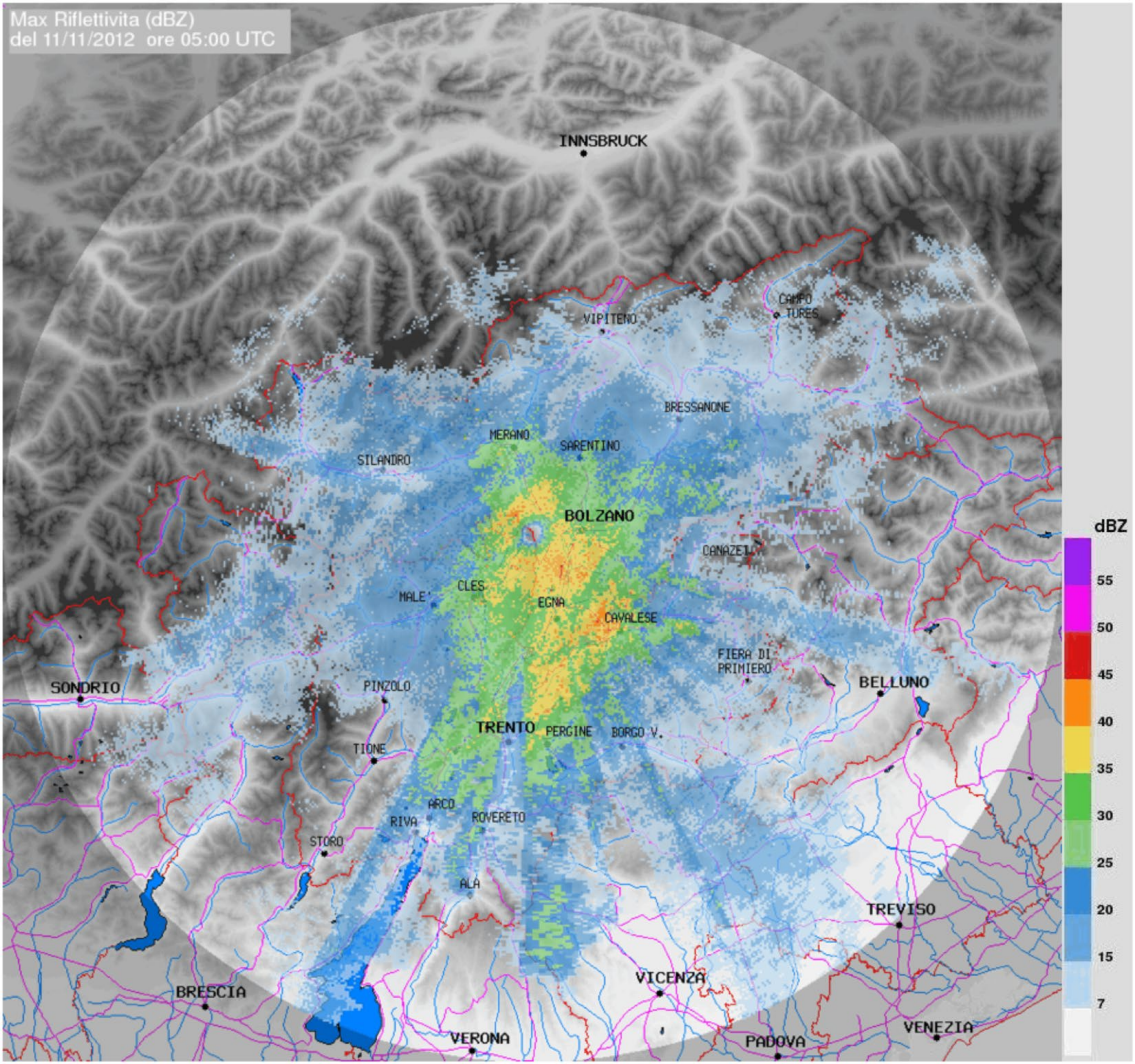
Example of the radar reflectivity (MAX(*Z*) product) in TAASRAD19.

**Fig. 4:**
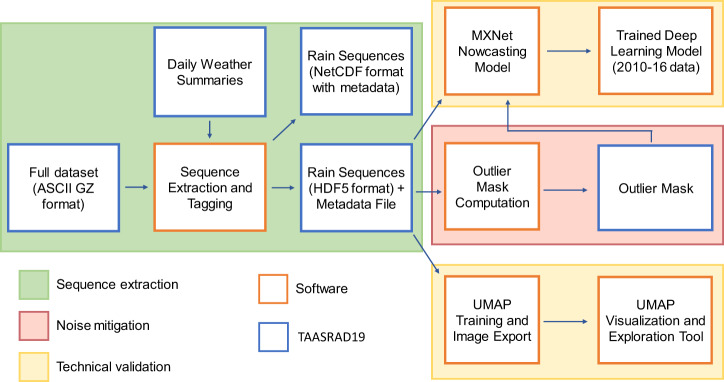
Overview of software methods for pre-processing and modeling of TAASRAD19 data.

**Fig. 5:**
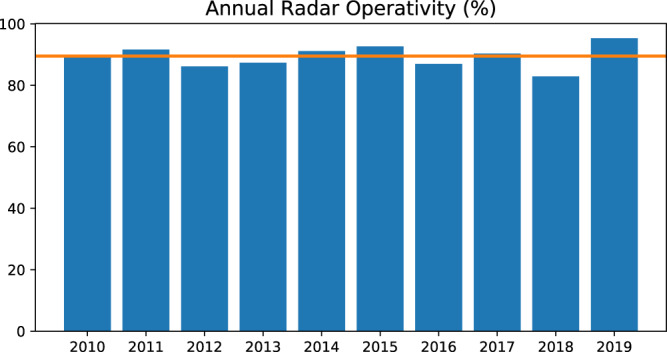
Radar operativity. Bar length represents the amount of time the radar has been in operation (not in maintenance or shut down), as percentage of valid 5 min frames over the total feasible in the year; yellow line: mean radar operativity (89.55).

Offline calibration of the radar system is performed at least once a year with scheduled maintenance for the calibration of both the transmitter and receiver ends. During normal operations, continuous monitoring on the receiver end is performed by the Built In Test Equipment (BITE), while polar volume quality assessment is performed as part of the regular scan strategy by monitoring variations of the recorded background noise at high elevation (long PTR scan at high elevations) and by solar scans. The polar reflectivity generated by the scan is filtered and corrected from the back-scattering of most of fixed obstacles using a Doppler correction filter^27^. The products used here are calibrated by the data provider Meteobolzano by taking into account the corrections computed as a results of the monitoring and calibration.

Although multiple data products can be derived by the radar, in this data descriptor we focus on the 2D maximum reflectivity MAX(*Z*) generated from the filtered polar volume provided to the authors from Meteobolzano. The MAX(*Z*) product is computed as the maximum value (expressed in dBZ) for each pixel of a predefined grid, measured on the vertical section in the filtered and corrected polar volume. For the Mt. Macaion radar, the product consists of a bi-dimensional metric grid of 480 × 480 pixels (projected in UTM 32 N coordinate system), with 500 m pixel size covering an area of 240 km of diameter (27,225 sq km) and centered at the radar site.

Despite the maximum reflectivity is potentially affected by noise, MAX(*Z*) is still preferred in the case of mountainous environments over alternative features commonly used for quantitative precipitation estimation, e.g. the *Constant Altitude Plain Position Indicators* (CAPPI). In fact, due to the high operating altitude of the receiver, CAPPI and similar products can miss precipitation events at altitudes lower than the radar location. Moreover, the MAX(*Z*) products helps alleviating the severe beam blockage that the radar experiences by the nearby mountains at lower elevation scans. Computation of the occlusion experienced by the radar at the first three elevations (0, 1 and 2 degrees) of the volume is displayed in Fig. 2, along with an example cross section. Note that MAX(*Z*) has been recently adopted as the standard by the pan-european radar composite for real time assessment purposes^28^.

The MAX(*Z*) product is thresholded at a lower bound of 0 dBZ: while the receiver can indeed observe a certain amount of drizzle in the negative range of MAX(*Z*), its detection is exceptionally uneven for the mt. Macaion radar, being drizzle mainly a low altitude phenomena. The high altitude of the receiver (1870 m.a.s.l.) and the severe beam blockage at low elevations allow the receiver to register drizzle almost exclusively in the underlying Adige valley, situated below the radar (200 m.a.s.l.). Even there, most of the drizzle is observed several hundred meters above the ground, and as such, it evaporates before reaching the ground. Given all these considerations and the specificity of the MAX(Z), it has always been standard practice for Meteotrentino to threshold the product at 0 dBZ.

An example of MAX(*Z*) product overlaid on the digital elevation model (gray colour map), main idrography (in blue), and administrative borders (red line) is displayed in Fig. 3.

In order to standardize the development of nowcasting models from TAASRAD19, both the original MAX(*Z*) products and the processed version of the dataset are available. For reproducibility, the methods used for pre-processing, modeling and validation are also provided. They are composed of three main sections:

### Sequence extraction

Obtaining contiguous and labelled precipitation sequences from the full image repository;

### Noise mitigation

Reducing noise and systematic artefacts in the MAX(*Z*) product;

### Technical validation

Deep learning forecasting and UMAP analysis.

In particular, the methods can be combined into a pipeline for developing nowcasting applications, with emphasis on those applying deep learning models. An overview of software methods for TAASRAD19 is displayed in Fig. 4; details for each main software module are provided in the following subsections.

## Sequence Extraction

The elementary patterns for training and operating nowcasting systems are sequences of radar time steps (frames). The sequence extraction process applied to the raw TAASRAD19 data is based on four basic requirements:

1. Each sequence must be contiguous in time (no missing frames), of sufficient length (at least two hours per sequence, to account for operational requirements of nowcasting methods and to guarantee sufficient decorrelation time^29^);
2. Each sequence should include at least one frame precipitation; sequences without precipitation signal are removed;
3. The full set of sequences should match the original data distribution in terms of seasonal occurrence (day/night, months, seasons), as well as precipitation types;
4. The sequences should be as clean as possible from noise or artefacts.

Descriptive statistics on the original data are listed in Table 2. The mean pixel value per frame varies from a minimum of 4.5 · 10^−4^ to a maximum of 32.3. Clearly, a positive minimum indicates the presence of noise in images, even in the absence of precipitation. A noise-mitigation strategy is thus needed. Figure 5 reports the annual radar operativity, i.e., the amount of time the radar has been in operation over the ten years (thus, not in maintenance or shut down), expressed as the percentage of valid 5 min frames over the total feasible in the year.

**Table 1:** Technical characteristics and operational parameters of the mt. Macaion radar.

Parameter	Value
Operational range	120 km
Maximum range	480 km
Resolution in range	250 m
Antenna Gain	45.8 db
dB beamwidth	0.9°
Wavelength	5.3 cm (5.6 Ghz)
Peak Power	307 kW
Pulse duration	0.8 *μ*s
Clutter to Signal Ratio (CSR)	8.0
Signal Quality Index (SQI)	0.25
Ground clutter correction	Doppler filter

**Table 2:** Descriptive Statistics of Radar frames included in TAASRAD19.

Total number of time steps	894,916
Total number of recorded days	3,292
Minimum – Maximum pixel values	0–52.5
Minimum – Maximum frame mean pixel values	4.5 · 10^−4^–32.3
Radar operativity between Jun 2010 and Dec 2019	89.55%

**Table 3:** Keywords used to extract the weak labels from the daily weather summaries.

Keyword	weak-label
precip, piov, piog	rain
grand	hail
temporal	storm
rovesc	downpour
nev	snow

Keywords correspond to word stems (in Italian) to account for plurals and other morphological inflections.

In addition to radar products, we collected the daily weather summary written by an operational meteorologist for each day. The summaries are provided in the form of a short overview, in Italian, describing the main meteorological conditions in the region during the day. A set of keywords corresponding to specific meteorological events (e.g. *storm, rain, snow, hail*) were extracted automatically from the summaries to tag the precipitation patterns from the radar sequences by *weak-labels*, i.e. labels that should be considered incomplete, inexact and inaccurate but are nonetheless useful for machine learning purposes^30^. The annotations in TAASRAD19 can be used in supervised or semi-supervised machine learning algorithms. The absence of those keywords has been combined with other descriptors of the radar images to identify and exclude sequences without precipitation events. The complete text of daily weather summaries are also released together with the radar data in the TAASRAD19 repositories^18,19^.

In summary, the sequence extraction process is composed of four steps:

### Data selection

To avoid seasonal imbalance, we select the interval 2010-11-01 and 2019-10-31, corresponding to exactly 9 years of data.

### Data chunking

The set of time steps is partitioned into multiple chunks of contiguous frames within a single day. Since some frames might be missing due to radar’s fault or errors in the processing pipeline, multiple chunks can account for the same day. Moreover, only chunks longer than 2 hours (i.e. 24 frames) are retained. Thus the length of each sequence varies from 25 to 288 contiguous frames, i.e. a single whole day with no missing data.

### Sequence filtering

**Fig. 6:**
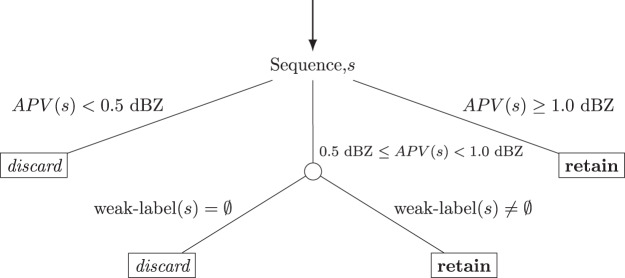
Decision tree of the strategy used to filter frame sequences based on corresponding APV value (the mean value over all pixels of the sequence), and *weak-labels*.

Sequences with no or few precipitation events are removed. To retain useful chunks, we adopt a selection strategy based on the *Average Pixel Value* (APV) of the chunk (defined as the mean value over all pixels of the sequence), and the *weak-labels* assigned to the corresponding day. First, all the sequences *s* where APV(*s*) < 0.5 dBZ are immediately discarded, whereas those with APV(*s*) > 1.0 dBZ are retained. We thus filter out sequences with only background noise and retain those with at least one precipitation pattern. For all the remaining sequences (i.e. 0.5 dBZ ≤ APV(*s*) ≤ 1.0 dBZ), we leverage on the *weak-labels* annotated from the daily summaries to identify sequences with precipitation events. Sequences with no label - *i.e.* with no precipitation event registered for the corresponding day - are discarded. A graphical representation of the decision strategy workflow is depicted in Fig. 6.

**Fig. 7:**
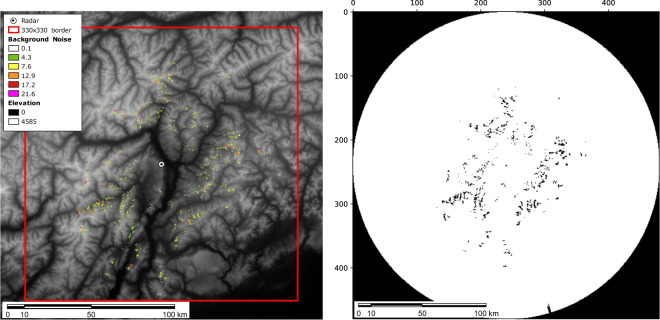
The left figure shows the map of noisy pixels and orography in the operation region; noise value is computed as the average of radar signal over the most clear sky images, showing systematic artifacts. The right image shows TAASRAD19 outlier mask; black pixels correspond to outliers and the area outside the radar operational range.

**Fig. 8:**
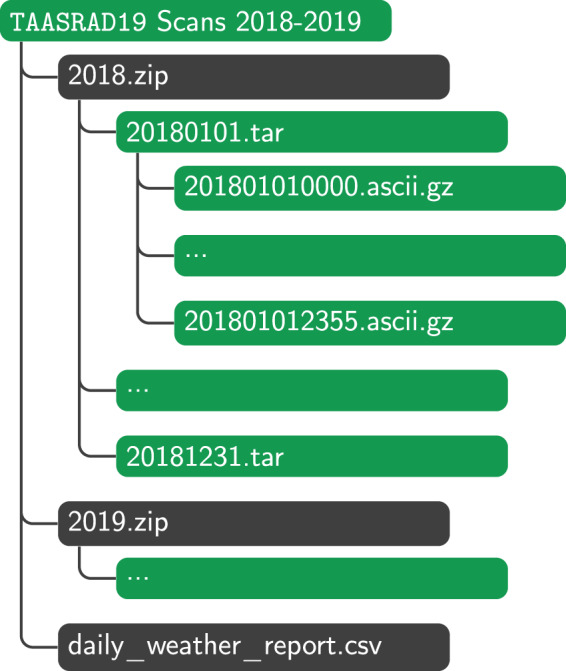
File structure of the TAASRAD19 ASCII image archive in the 2018–19 repository.

**Fig. 9:**
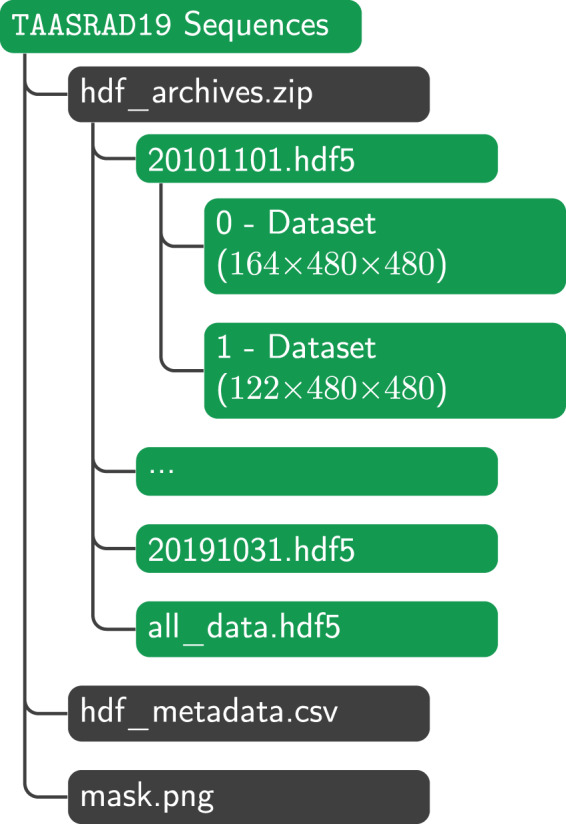
File structure of the TAASRAD19 HDF5 sequence archive.

**Fig. 10:**
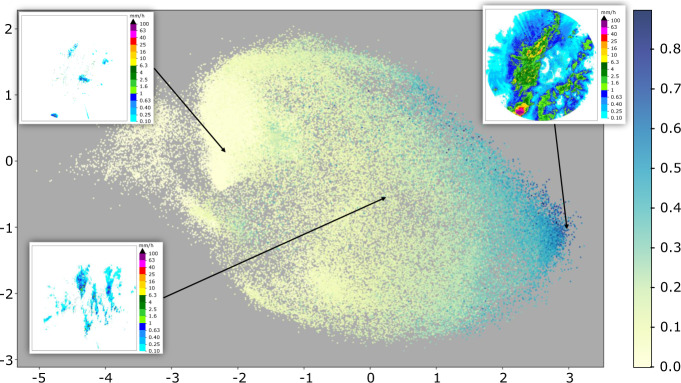
UMAP Embedding for TAASRAD19_u162k: plot of the first (x axis) and third (y axis) components. Each point is a radar frame in the projected UMAP space, colorized by Wet Area Ratio (WAR). Frames with similar rain patterns are placed closer together. Insets show examples of three different precipitation patterns and their position in the UMAP projected space.

**Fig. 11:**
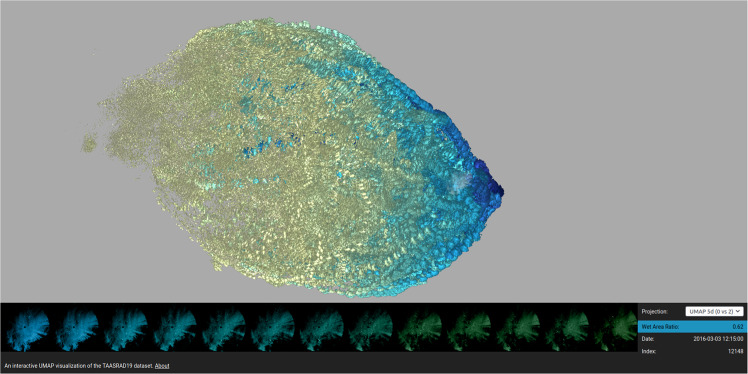
Screenshot of the interactive radar analog exploration tool.

### Sequence labelling

All the retained sequences are labelled according to the corresponding *weak-label* from the daily summary, wherever possible. The complete list of all keywords used (in the form of word stems, in Italian), and corresponding *weak-labels* is reported in Table 3.

The resulting number of sequences in TAASRAD19 is 1,732, describing a total of 362,233 time steps, mapped to 1,258 days of precipitation data. Sequences are available at the Zenodo TAASRAD19 repository^20^, along with related metadata files, including labels and statistics.

## Noise Mitigation

The main goal of the noise removal step is to identify recurring noise patterns (i.e. outliers in specific pixel location) that can consistently occur in most radar images. Removing such outliers is particularly important, especially for methods (e.g. machine learning) whose performance may be affected by the presence of values (largely) out of the data distribution. We investigate the issue by generating a map to observe the presence of outlier pixels, from which we then derive a data-driven strategy for a *global outlier mask*.

### Noise analysis

To check for outlier pixels, we ranked each time step for increasing APV (i.e. from the least to the most rainy) and we considered the top 0.1% of the ranking (895 frames) to compute a map of background noise. The noise map was generated as the average (per-pixel) of the 895 less rainy frames, which correspond to clear sky condition sampled at different times through the dataset.

As shown in Fig. 7, where the computed map is overlaid to the digital terrain model, there is thus evidence of systematic artifacts in the signal.

### Outlier mask

Systematic noise signals can be associated to non-fixed structures, e.g. clutter, multipath returns, or several other effects. In the case of the mt. Macaion radar, most of the noise still present in the data product is due to moving objects sensed on the terrain surface (e.g. trees moving during high wind days). The objective is thus to build a mitigation technique aimed at reducing the impact of high value noise in localized pixels present in most dataset operating days, thus managing possible non-meteorological moving artefacts on the ground. In order to filter the noise in the frames, a *global outlier mask* can be generated based on a distance measurement between distributions of pixel values over time (Fig. 7). We construct this mask using the *Mahalanobis distance*, as in^31^. In details, first the distribution histogram of the pixel values over a random sample (20% of the sequences) is computed by binning the ratios of pixel value in a location *i* in *N* = 526 bins *x*_*i*_ (each bin corresponding to a step of 0.1 dBZ). Then, we extract the corresponding sample mean

$$\widehat{\mu }=\frac{1}{N}\mathop{\sum }\limits_{i=1}^{N}{x}_{i}$$

and covariance matrix

$$\widehat{S}=\frac{1}{N-1}\mathop{\sum }\limits_{i=1}^{N}({x}_{i}-\mu )({x}_{i}-{\mu }^{T})$$

to evaluate the Mahalanobis distance of *x* as

$${D}_{M}=\sqrt{{(x-\widehat{\mu })}^{T}{\widehat{S}}^{-1}(x-\widehat{\mu })}$$

where ${\widehat{S}}^{-1}$ is derived by using the Moore-Penrose pseudoinverse. Pixels that have a Mahalanobis distance higher than the mean distance plus three times the standard deviation are marked as outliers. We finally obtain a binary mask with 179,333 inliers. Excluded pixels are 1,627 outliers and 49,440 points outside the radar operation range of 120 km (equivalent to a 240 pixel radius from the Mt. Macaion site). The TAASRAD19 outlier mask is mapped in Fig. 7. Notably, the binary mask can also be used to skip calculation on the masked pixels when computing the loss function in deep learning models. The TAASRAD19 outlier mask is also available as binary PNG file in the Zenodo repository^20^.

## Data Records

TAASRAD19 is available on Zenodo, split in four repositories to comply with data size limits. The full MAX(*Z*) raw data archive is organized in two different repositories, one for years 2010–2016^18^ and another one for years 2017–2019^19^, while the sequences are available at^20^ and^21^, respectively. The product archive is organized by acquisition time for an easier automatic processing, using a three-level structure represented in Fig. 8. The organization of the data retains the hierarchy originally provided by Meteotrentino: this decision is motivated by the aim to provide a fully reproducible end-to-end data generation pipeline that can be run starting from the original raw dataset. Frames recorded the same year are archived together in a single ZIP file; each day of the year is archived in a single TAR files containing radar scan compressed using the GZIP algorithm to reduce disk space. A CSV file with the daily weather summaries (i.e. daily_weather_report.csv) for all 9 years is also available, replicated in the two product repositories.

The data hierarchy has been designed to facilitate training machine learning models. The experimental setup of a classification/regression task usually requires an extensive number of repeated runs where data is supplied to the learning algorithms in small chunks, whose corresponding archived batches can be decompressed at runtime, thus optimizing the operational flow. In this scenario, the per-year organisation of the archives for the raw data is preferred over the unique monolithic alternative to allow users to set a preferred training strategy for training and testing over specific time intervals within data analysis plans, also considering appropriate cross-validation procedures. The configuration is indeed used in the analysis. For example, practitioners usually find it convenient to keep the elementary batch file compressed, thus we keep the basic blocks as zip files. One level up, an organization for yearly time steps allows accounting for seasonalities, also maintaining large flexibility. The data structure in our case, including four different repositories, 2 reps for the raw data and 2 reps for the sequences, each split in one rep for the years 2010–2016 and one for the years 2017–2019 enables an easy generation of training/validation/test partitions in Machine/Deep Learning settings.

The text files follow the GRASS ASCII Grid format (https://gdal.org/drivers/raster/grassasciigrid.html and https://grass.osgeo.org/grass78/manuals/r.in.ascii.html), which is a plain-text, human readable grid representation: each row in the file represents a row in the grid, where each cell value is a floating point number separated by a tabulation character. An optional nodata value (representing NULL value) can be specified: by convention, in TAASRAD19, the NULL data value is −99, and in our workflow it is converted to 0 upon parsing the files. The format can be parsed in any programming language without the need of specific software packages; geo-reference information is included in a header before the data rows. This structure allows a seamless data loading into Geographical Information Systems (GIS) software suites, e.g. QGIS^32^, GRASS^33^. It also facilitates the conversion of the product in different formats of choice by libraries such as GDAL^34^.

However, even if the compressed ASCII format is extremely easy to create and manipulate programmatically, it is very inefficient in terms of throughput, and processing power required for data ingestion. Moreover, metadata and other attributes cannot be incorporated using this data format.

For these reasons, the extracted sequences are made available in both HDF5^35^ and NetCDF^36^ format, that are both widely used in meteorological applications^37^. Conversion between the two formats can be easily obtained by well established libraries such as *xarray*.

The HDF5 release^20^ is mainly aimed at supporting a straight integration of the dataset into modeling pipelines, thanks to the large support of the format in many machine learning platforms, and in the majority of scientific environments. Notably, HDF5 is the format of choice for many Deep Learning (DL) frameworks, that offer native support (e.g. Keras, TensorFlow), or straightforward integration hooks (e.g. PyTorch, mxnet/Gluon) for HDF5 datasets. Sequence data in the HDF5 release is organized similarly to the image archive (see Fig. 9). Sequences from the same day are saved together in a single HDF5 file named after the date of the day. A file named all_data.hdf5 stores links to all the daily files, and can be used to iterate over all the sequences. The whole HDF5 archive is stored on Zenodo on a single ZIP file (i.e. hdf_archives.zip). The minimum hdf5 library version to read the files is 1.10.4. Two other files are available: a PNG image (mask.png) representing the pre-computed outlier mask, and a CSV file (hdf_metadata.csv) with relevant metadata about each sequence. Metadata include: id of the sequence, start and end time, sequence length, average pixel value, corresponding weak-labels extracted from the daily weather summaries (if any).

The NetCDF release^21^ is aimed at maximum compatibility with existing meteorological and climatological tools for data analysis and exploration. The dataset mimics the same file structure of the HDF5 release (one file per day) and is further supplemented by extensive metadata (e.g. reference coordinates, data types, date/time reference, daily tags, sequences length). To ensure maximum compatibility, a flattened structure is used and NetCDF4 groups are avoided. Sequence lengths and date-time attributes are both reported in metadata, and can be used to determine the start and end frame of each sequence. The produced format follows the Climate and Forecast (CF) Metadata conventions and has been validated for the use with compatible tools using the CF-Checker suite (https://github.com/cedadev/cf-checker) against the CF-Convention 1.7 standard.

## Technical Validation

We outline here two examples of deep learning and analytical applications in meteorology and precipitation forecasting based on TAASRAD19.

### Deep learning for precipitation nowcasting

Analog ensemble models^26,38,39^ or extrapolation methods^12^ are mainly used for probabilistic forecasting; however convolutional recurrent neural networks are now the state of the art for deterministic nowcasting^31,40–43^. In^22^ we used TAASRAD19 to train a deep learning model that forecasts reflectivity up to 100 min ahead (i.e. 20 frames) at full spatial spatial resolution of the radar (0.5 × 0.5 km), based on 25 min (i.e. 5 frames) of input data. The model is an evolution of^41^, based on the TrajGRU architecture, described in^31^. A Python implementation using the Apache MXNet^44^ deep learning framework is available at https://github.com/MPBA/TAASRAD19 (for the original version see https://github.com/sxjscience/HKO-7).

In our experimental setup, TAASRAD19 sequences extracted from June 2010 to December 2016 are used for training, whilst the model is tested in inference on sequences from 2017 to 2019. Training and validation sequences are extracted with a moving-window strategy applied along the entire set of contiguous sequences included in TAASRAD19. The generated sub-sequences are 25 frames long, where the first 5 frames are used as input, and the remaining 20 ones are used as ground truth for validation. In summary, 220,054 and 122,548 sub-sequences have been generated for *training* and *validation*, respectively.

To allow a fair comparison with results reported in^31^ on the Hong Kong (HKO-7) dataset, we implement the same model hyper-parameters: the model is trained for 100,000 iterations considering a batch size of 4, using two NVIDIA GTX1080 GPUs in parallel, with 8 GB of memory each. Network weights for our trained model are available on GitHub. We evaluate results using the Critical Success Index (CSI) score, a metric commonly used in computational meteorology, as defined in^31^: output predictions and ground truth frames are first converted to rain rate using the Marshall-Palmer Z-R relationship^8^, then binarized at different thresholds to test model performance over different rain regimes. Results on the validation data set are reported in Table 4. Scores for both models are satisfactory for potential application as a score of CSI > 0.45 (for *r* ≥ 0.5) means that the model is reliable for predicting precipitation occurrence. Results reported for the HKO-7 dataset are consistently better; disregarding the use of the MAX(*Z*) product instead of CAPPI as inputs, differences are expected due to the higher variability of Alpine landscape and the different spatial resolutions (0.5 km for TAASRAD19 vs. 1.07 km for HKO-7).

**Table 4:** Critical Success Index (CSI) scores for TrajGRU on TAASRAD19 and in Shi *et al*.^31^, on HKO-7 dataset; *r* is the instantaneous rain rate (mm/h).

Model and Dataset	*r* ≥ 0.5	*r* ≥ 2	*r* ≥ 5	*r* ≥ 10	*r* ≥ 30
TrajGRU on HKO-7^31^	0.553	0.476	0.375	0.284	0.186
TrajGRU on TAASRAD19	0.487	0.283	0.190	0.144	0.078

### Analog exploration by UMAP embedding

The search for analogs, i.e. similar weather patterns in the past, is a key approach in meteorology. It usually requires to perform a fast and accurate query for similar spatio-temporal precipitation patterns in very large archive of historical records.

In^26^, we introduced a framework for fast approximate analog search retrieval of radar sequences that employs a two-step process dimensionality reduction and fast similarity search to improve accuracy and computational performance. The framework combines Mueen’s Algorithm for Similarity Search^25^ (MASS) with the Uniform Manifold Approximation and Projection (UMAP) algorithm^23^. UMAP resulted more effective as a dimensionality reduction technique for radar images, in combination with MASS, than the standard Principal Component Analysis (PCA)^26^.

Here we leverage UMAP dimensionality reduction features for the interactive visualization of radar images from the TAASRAD19 dataset. To realize a real-time interaction on a massive sample of images, we first pre-processed all HDF5 sequences by resizing the images from 480 × 480 to 64 × 64 pixel using bi-linear interpolation. Normalization between 0 and 1 is obtained by dividing each pixel value by 52.5, i.e. the maximum reflectivity value supported by the radar (see Table 2). The first 200,000 images (out of 362,233) are used as training data for a UMAP model with the following hyper-parameters: neighbors = 200; components (dimensions) = 5; min-distance = 0.1; metric = *euclidean*. The UMAP algorithm outputs a dimensionality reduction map (from 64 × 64 = 4,096 to 5), which distributes images in the reduced space by preserving the reference distance metric as in the original space (Euclidean, in this case). Given that Euclidean distance is rank preserving with regard to mean squared error, similar precipitation patterns result closer in the reduced space. In Fig. 10 we show an example of UMAP planar embedding of the remaining 162,233 frames (TAASRAD19_u162k), where each point is coloured by Wet Area Ratio (WAR), defined as the percentage of pixels in the frame with a rain rate higher than 0.1 mm/h. Examples of different precipitation patterns in TAASRAD19_u162k are shown as insets within the figure. From left to right (UMAP component 1), locations in the projected space correspond to patterns of increasing WAR.

The approach has been engineered as UMAP Radar Sequence Visualizer, a tool for interactive exploration of sequence analogues in radar archives. Sets of radar sequences can be imported for visualization in an interactive web canvas built as React/NodeJS application, derived from the UMAP Explorer tool (https://grantcuster.github.io/umap-explorer/).

Each radar frame is placed as a mini image on the explorable canvas based on its coordinates in the UMAP projection. The canvas can be panned and zoomed, and each image is colored by WAR using a yellow-to-blue gradient. When an image is selected, the lower panel shows the next images in the sequence, highlighting the evolution of the precipitation pattern. Projections over different UMAP axis pairs can be selected. The source code of the tool, along with scripts and examples on how to export data for visualization, are available in the GitHub repository.

An online demo of the UMAP Radar Sequence Visualizer is also available (reachable from the TAASRAD19 GitHub repository). The online dashboard is currently equipped with TAASRAD19_u50k, a sample of moderate size (50,000 images for 18 months of observations) to allow browsing with limited RAM resources (Fig. 11). In the online tool, frames with precipitation concentrated in the Northern part of the region are also located on the upper area of the plot, while those with rain in the South lie on the lower part of the plot. Blue images on the rightmost sector of the plot represent extreme events, while scattered points on the left of the main cloud correspond to less severe precipitation patterns.

## Usage Notes

When using the frames or the sequences in machine learning workflows, we suggest to set no-data cells (value −99.0) to 0 and normalize the data in $[0,1]$ interval by dividing by 52.5 before feeding the batch of data to the model; whilst for computer vision applications it can be useful to transform the frames to grayscale images by applying a lossy conversion to 8-bit integers (values from 0 to 255).

## Data Availability

All the software described in Technical Validation is available in a public GitHub repository (https://github.com/MPBA/TAASRAD19), along with the Python scripts for sequence pre-processing, installation scripts for the MXNet^44^ framework, pre-trained network model weights, and examples of radar prediction output sequences. All the code was written in Python 3.6 and tested on Ubuntu releases 16.04/18.04. Some pre-processing steps (e.g. sequence and outlier mask generation) require a non trivial amount of computing resources and memory. Training the deep learning model with the same parameters described in the paper requires either two GPUs with 8GB of RAM or one GPU with 16GB. Please refer to the README files in the code release for further instructions.
